# F-waves in acute sciatic pressure palsy

**DOI:** 10.4103/0972-2327.42944

**Published:** 2008

**Authors:** S. Raghavendra, V. Vibhin, H. K. Anand

**Affiliations:** Department of Neurology, Kanva Diagnostic Centre, Bangalore, India; 1 Department of Radiology, Kanva Diagnostic Centre and ^1^Clumax MRI Centre, Bangalore, India

A 57-year-old gentleman presented with acute onset weakness and paresthesia of the distal right lower limb, of one day duration. On the previous day, he continuously was on a chair consuming alcohol. He had a history of localized low back pain for about a year, which had not currently aggravated. There were no bowel or bladder symptoms. Examination showed weakness of the right foot (dorsiflexion 4/5, plantar flexion 4/5) and knee flexors (4/5). Knee extensors and hip power, including abductors, were normal. Right ankle reflex was absent. Other deep tendon reflexes were normal. Right plantar response was mute and left was flexor. Sensory examination showed no abnormality. Straight leg raising (SLR) test was negative bilaterally.

Nerve conduction showed normal distal latencies and CMAP amplitudes from the lower limb nerves [Table 1]. F-wave study showed normal persistence and normal Fm (minimum) latencies from the lower limb nerves. F-waves from the right tibial and peroneal nerves showed reduced amplitude with increased chronodispersion (Fc) [Figure 1A,B]. H-reflexes was absent from the right soleus; and was normal from the left.

**Table 1:** Nerve conduction parameters in a 57-year-old gentleman with acute onset of weakness of the right lower limb, due to sciatic pressure palsy

Nerve	Distal latency (ms)	Amplitude (mv)	Conduction velocities (m/s)	F min (ms)	Fc (ms)
Right peroneal	5	10.4	47	50.4	8.6
Left peroneal	4.7	14.9	45	51	4.5
Right tibial	4.7	16.1	44	47.3	14.7
Left tibial	4.3	15.5	45	49.9	5
Right femoral	3.4	6.5	-	-	-
Left femoral	3.5	6.1	-	-	-
Right sural*	3.9	12	43	-	-
Left sural*	3.2	10	47	-	-

**Figure 1:**
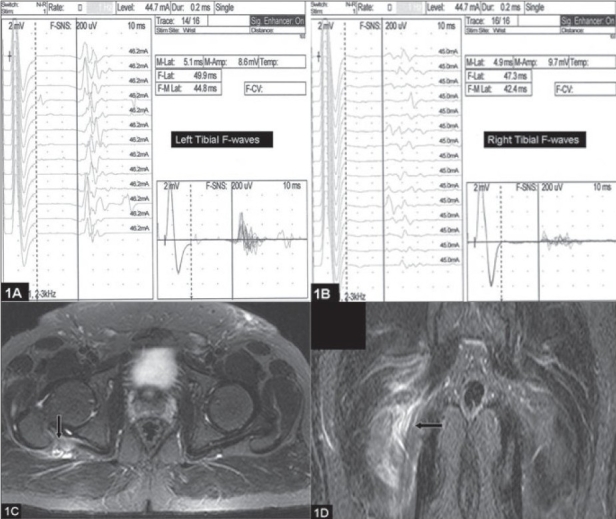
A, B, demonstrates reduced amplitude and increased chronodispersion (Fc) of the right tibial F-waves. Axial T1 (C) and Coronal T2 W IRFSE (D) sequences through pelvis shows thickened edematous right sciatic nerve with surrounding soft tissue edema (arrow)

An MRI examination showed right sciatic nerve edema and of the surrounding soft tissue [Figure 1C, D]. MRI of the lumbosacral spine was normal. The diagnosis of right sciatic nerve pressure palsy was made.

The patient was treated with one gram intravenous methylprednisolone daily for four days, followed by oral steroids. He recovered completely in two weeks, except for absent right ankle jerk.

## Discussion

This utility of H-reflex and F-wave parameters in the assessment of a proximal lesion with normal distal conduction parameters is well-depicted ( an acute sciatic neuropathy here). Weber *et al.*[1] have reported utility of Fc to be twice as Fm in the lower limbs in this situation.
